# Interleukin‐23 receptor defines T helper 1‐like regulatory T cells in oral squamous cell carcinoma

**DOI:** 10.1002/iid3.746

**Published:** 2022-11-23

**Authors:** Wei Li, Ning An, Mingwei Wang, Xiguo Liu, Zhidan Mei

**Affiliations:** ^1^ Department of Head and Neck Surgery, Hubei Cancer Hospital, Tongji Medical College Huazhong University of Science and Technology Wuhan China; ^2^ Department of Pathology, Hubei Cancer Hospital, Tongji Medical College Huazhong University of Science and Technology Wuhan China

**Keywords:** interleukin‐23 receptor, oral squamous cell carcinoma, regulatory T cells, T helper 1 cells, tumor immunity

## Abstract

**Background:**

The immune responses play significant roles in the onset, progression, and outcome of oral squamous cell carcinoma (OSCC). CD4^+^ regulatory T cells (Tregs) significantly impact tumor immunity. However, their role in OSCC development remains elusive.

**Methods:**

In a carcinogen‐induced mouse OSCC model, interleukin‐23 receptor (IL‐23R) expression on Tregs and Treg function were determined by flow cytometry. IL‐23R overexpression in Tregs was achieved by lentiviral infection, followed by evaluation of the expression of Forkhead box P3 (Foxp3), T‐bet, retineic‐acid‐receptor‐related orphan nuclear receptor gamma t, and cytokines by flow cytometry. Adoptive transfer assays were applied to analyze the function of IL‐23R^−^overexpressing Tregs in vivo. The cellular sources of IL‐23 were also determined by flow cytometry.

**Results:**

IL‐23R^−^ Tregs and IL‐23R^+^ Tregs were found in the tongues but not spleens of OSCC‐bearing mice. IL‐23R^+^ Tregs expressed lower Foxp3 but higher T‐bet than IL‐23R^−^ Tregs. IL‐23R^−^ Tregs produced abundant IL‐10 and transforming growth factor (TGF)‐β, while IL‐23R^+^ Tregs produced lower IL‐10 and TGF‐β but remarkably higher interferon (IFN)‐γ. Furthermore, IL‐23R^+^ Tregs possessed more phosphorylated signal transducer and activator of transcription (STAT3) and STAT4 than IL‐23R^−^ Tregs. IL‐23R^+^ Tregs were less immunosuppressive than IL‐23R^−^ Tregs, as evidenced by weaker inhibition of activated conventional T cells. IL‐23R overexpression in splenic Tregs remarkably reduced the expression of IL‐10 and TGF‐β but increased IFN‐γ expression when Tregs were adoptively transferred into OSCC‐bearing mice. In the OSCC microenvironment, macrophages, dendritic cells, and malignant OSCC cells produced IL‐23 which might modulate the function of IL‐23R^+^ Tregs.

**Conclusions:**

This study unveils Treg heterogeneity, thus deepening the understanding of Treg biology and tumor immunity in OSCC.

## Graphical abstract

interleukin‐23 receptor (IL‐23R) is differentially expressed in infiltrating Tregs in a mouse oral squamous cell carcinoma model. IL‐23R‐ Tregs are immunosuppressive Tregs while IL‐23R + Tregs have Th1 properties.

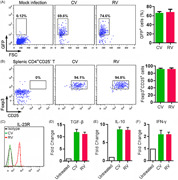

## 1 INTRODUCTION

Oral squamous cell carcinoma (OSCC), featuring squamous cell‐derived neoplasm in the oral cavity, is the most frequent malignancy of head and neck cancers.^1^ The immune system controls the onset and progression of OSCC.^2^ Tumor‐infiltrating lymphocytes in OSCC, including natural killer cells, B cells, and T cells play significant roles in the anti‐OSCC immunity.^3^, ^4^ Among the tumor‐infiltrating effector T cells, regulatory T cells (Tregs) which express Forkhead box P3 (Foxp3), are critical for maintaining immune homeostasis and tolerance and they suppress antitumor immunity. OSCC patients are characterized by an increase in the number of circulating and infiltrating Tregs.^5^, ^6^ However, the effect of Tregs in OSCC remains controversial. In a recent study using a mouse model of carcinogen‐induced OSCC, Treg ablation exacerbates OSCC.^7^ Nonetheless, previous studies suggested that increases in circulating or infiltrating Tregs were associated with poor prognosis, whereas other research proposed the opposite conclusion.^8^ Therefore, the exact role of Tregs in OSCC development has not been thoroughly elucidated.

Tregs exhibit profound phenotypic and functional heterogeneity. Under various circumstances, Tregs gain the expression of transcription factors and cytokines normally associated with Th1, Th17, and other T helper subsets.^9^, ^10^ Treg heterogeneity in cancers has also been reported. Th1‐like Tregs coexpressing T‐box expressed in T cells (T‐bet) and Foxp3 are identified in the tumor tissues of human hepatocellular carcinoma.^11^ Th1‐like Tregs also exist in a mouse lung carcinoma model and human non‐small cell lung carcinomas.^12^ Th17‐like Tregs co‐expressing Foxp3 and IL‐17 might support the development of colorectal cancer and ovarian cancer.^13^, ^14^ Th2‐like Tregs, which express Th2‐associated cytokines, are present in malignant tissues of patients with melanoma and colorectal cancer.^15^ Treg heterogeneity possibly impacts the prognosis of cancer patients because it could modulate tumor growth, survival, and metastasis.^16^ However, whether Treg heterogeneity is present in OSCC remains unknown, not to mention the potential effects of heterogeneous Treg populations on OSCC pathogenesis.

Interleukin (IL‐23) is an IL‐12‐related proinflammatory cytokine comprising two subunits, IL‐23 p19 and IL‐12/23 p40.^17^ It is mainly secreted by activated macrophages and dendritic cells.^17^ It binds to the interleukin‐12 receptor β1 chain and requires IL‐23 receptor alpha (IL‐23Rα) to transduce signaling that activates transcription activator signal transducer and activator of transcription (STAT3), STAT4, and Janus kinase 2 (JAK2).^18^ The IL‐23/IL‐23R axis is involved in the maintenance of Th17 cells.^19^ IL‐23 could drive the generation of Th17‐like Tregs in psoriasiform dermatitis and leprosy.^20^, ^21^ Recent research also demonstrated the significance of IL‐23R in the differentiation and function of Th1 and Th2 cells.^22^, ^23^ Interestingly, IL‐23 has been reported to be expressed in human OSCC cell lines and OSCC tissues to promote OSCC growth.^24^, ^25^ Therefore, IL‐23, either macrophages‐derived or OSCC‐derived, might influence the function of OSCC‐infiltrating Tregs if they express IL‐23R. However, to our knowledge, this has not been reported by previous studies.

In this research, we revealed the differential expression of interleukin‐23 receptor (IL‐23R) in infiltrating Tregs in a mouse OSCC model. We further demonstrated that IL‐23R^‐^ Tregs were immunosuppressive Tregs while IL‐23R^+^ Tregs had Th1 properties. Therefore, our study unveils Treg heterogeneity and thus deepens the understanding of Treg biology in OSCC.

## 2 MATERIALS AND METHODS

### 2.1 Murine OSCC model

This animal research was reviewed and approved by Hubei Cancer Hospital. The experimental procedures were carried out under the Animal Research: Reporting of In Vivo Experiments (ARRIVE) guidelines. Male Foxp3‐eGFP transgenic mice (8‐week old, C57BL/6J background) and wild‐type C57BL/6J mice were purchased from Hunan SJA Laboratory Animal Co., Ltd. They were bred under a pathogen‐free environment with a 14‐h light/10‐h dark cycle and 40% humidity. Their autoclaved diet contained 6% fat and the bedding was changed every week.

The carcinogen 4‐Nitroquinoline 1‐oxide (4‐NQO, Sigma‐Aldrich, Cat# 56‐57‐5) was prepared in DMSO at 50 mg/ml and stored at −20°C before use. It was diluted in propylene glycol (Sigma‐Aldrich, Cat# 57‐55^−^6) and added into sterile water at a final concentration of 100 μg/ml. The mice were fed with drinking water containing 100 µg/ml 4‐NQO for 18 weeks, followed by drinking sterile water for additional 2 or 6 weeks before analysis (Supporting Information: Figure 1). The 4‐NQO‐containing water was replaced weekly.

### 2.2 Enrichment of mononuclear cells from tongues and spleens

The reagents were purchased from Sigma‐Aldrich. The mice were anesthetized with inhalant isoflurane. Each animal was intracardially perfused with 30 ml of ice‐cold 0.9% saline. After that, the tongues were excised and minced into ~1 mm^3^ pieces, followed by digestion in 1 ml of RPMI1640 medium (Cat# R8758) containing 5% fetal calf serum (FCS, Cat# F2442), 500 µg/ml collagenase IV (Cat# C4‐BIOC), 100 µg/ml DNase I (Cat# 10104159001), 5 mM EDTA (Cat# E9884), and 2.5 mM calcium chloride (Cat# C8106) for 20 min at 37°C. The digested tissues were gently ground in a 70‐µm nylon mesh and the resultant tissue homogenates were mixed with 4 volumes of 30% percoll (ThermoFisher, Cat# MP219536980) before being loaded onto an equal volume of 70% percoll. The tissue‐percoll mixture was centrifuged at 500*g* for 20 min. Mononuclear cells floating in the interface of 30% percoll and 70% percoll were collected and washed with ice‐cold saline for further experiments. Mouse splenocytes were prepared by gently pressing spleens through a 70‐µm nylon mesh. Red blood cells were lysed by incubating splenocytes in 1 ml of Tris‐NH_4_Cl buffer for 3 min at room temperature.

### 2.3 Isolation of total tongue cells

The isolation procedure was the same as the method for mononuclear cell enrichment, except that no percoll gradient centrifugation was performed.

### 2.4 Flow cytometry analysis and cell sorting

The antibodies used for flow cytometry were listed in Table 1. To stain cell surface proteins, 1 × 10^6^/ml cells were incubated with 5 µg/ml of each antibody on ice for 15 min before analysis on a BD FACSCalibur™ cytometer (BD Biosciences). If the primary antibody was unconjugated, the cells were further stained with PE goat anti‐rabbit IgG on ice for 15 min before analysis. For cell sorting, stained cells were enriched using a BD InFlux Cell Sorter (BD Biosciences). To stain intracellular cytokines, cells were stimulated with 50 ng/ml phorbol 12‐myristate 13‐acetate (PMA, Sigma‐Aldrich, Cat# P8139) and 1 μg/ml ionomycin (Sigma‐Aldrich, Cat# I9657) in the presence of 2.5 mg/ml brefeldin A (BioLegend, Cat# 420601) and 2 μM monensin (BioLegend, Cat# 420701) for 3 h. Cells were then fixed with 2% paraformaldehyde (Sigma‐Aldrich, Cat# P6148) for 20 min, permeabilized with cold 90% methanol‐phosphate‐buffered saline for 30 min, and incubated with 5 µg/ml of antibodies for 1 h at room temperature. Cell apoptosis was determined by incubating the cells with PE Annexin V (BioLegend, Cat# 640947) and 2 µg/ml propidium iodide (BioLegend, Cat# 421301) following the manufacturer's manual.

**Table 1:** Flow cytometry antibodies

Antibody	Cat#	Clone#	Vendor
Pacific Blue™ anti‐mouse CD3	100213	17A2	BioLegend
APC/Cyanine7 anti‐mouse CD4	100413	GK1.5	BioLegend
APC anti‐mouse IL‐23R (against IL‐23Rα)	150905	12B2B64	BioLegend
PE/Cyanine7 anti‐mouse IL‐10	505025	JES5‐16E3	BioLegend
PE anti‐mouse TGF‐β1	141403	TW7‐16B4	BioLegend
PE/Cyanine7 anti‐mouse IFN‐γ	505825	XMG1.2	BioLegend
PE anti‐mouse IL‐17A	506903	TC11‐18H10.1	BioLegend
PE/Cyanine7 anti‐mouse IL‐4	504117	11B11	BioLegend
PE anti‐mouse IL‐5	504303	TRFK5	BioLegend
PE anti‐mouse CD62L	161203	W18021D	BioLegend
PE/Cyanine7 anti‐mouse/human CD44	163607	QA19A43	BioLegend
APC anti‐mouse CD45	103111	30‐F11	BioLegend
PE anti‐mouse CD31	160203	W18222B	BioLegend
PE/Cyanine7 anti‐mouse CD11c	17317	N418	BioLegend
APC/Cyanine7 anti‐mouse F4/80	157315	QA17A29	BioLegend
APC anti‐mouse CD25	101909	3C7	BioLegend
PE anti‐mouse Foxp3	126403	MF‐14	BioLegend
PE/Cyanine7 anti‐mouse T‐bet	644823	4B10	BioLegend
PE anti‐mouse/human Ki‐67	151209	11F6	BioLegend
PE/Cyanine7 anti‐mouse RORγt	25‐6981‐82	B2D	ThermoFisher
PE anti‐mouse/human Phospho‐STAT3 (Tyr705)	12‐9033‐42	LUVNKLA	ThermoFisher
PE anti‐mouse/human Phospho‐STAT4 (Tyr693)	MA5‐37332	Stat4Y693‐F6	ThermoFisher
PE anti‐mouse/human Phospho‐STAT5 (Tyr694)	12‐9010‐42	SRBCZX	ThermoFisher
PE anti‐mouse/human Phospho‐STAT6 (Tyr641)	12‐9013‐42	CHI2S4N	ThermoFisher
Unconjugated anti‐mouse/human MCT4	22787‐1‐AP	Polyclonal	ThermoFisher
PE goat anti‐rabbit IgG	P‐2771MP	Polyclonal	ThermoFisher
Alexa Fluor® 488 anti‐mouse IL‐23 p19	53‐7023‐82	fc23cpg	ThermoFisher

Abbreviations: INF, interferon; RORγt, retineic‐acid‐receptor‐related orphan nuclear receptor gamma t; STAT, signal transducer and activator of transcription; TGF, transforming growth factor.

### 2.5 Quantitative reverse transcription and polymerase chain reaction (qRT‐PCR)

The reagents were purchased from ThermoFisher Scientific. Cellular RNAs were extracted using the Arcturus PicoPure RNA Isolation Kit (Cat# KIT0204). Complementary DNAs (cDNAs) were made using the RevertAid First Strand cDNA Synthesis Kit (Cat# K1622). cDNAs were mixed with the PowerUp™ SYBR® Green Master Mix (Cat# A25742) and quantitative PCR was carried out on a CFX Connect Real‐Time PCR Detection System (Bio‐Rad). Primer sequences are shown in Table 2.

**Table 2:** Primer sequence

Target	Forward (5′ to 3′)	Reverse (5′ to 3′)
IL‐10	aggcgctgtcatcgatttct	atggccttgtagacaccttgg
TGF‐β	ctgctgacccccactgatac	gtgagcgctgaatcgaaagc
IFN‐γ	cagcaacagcaaggcgaaaaagg	tttccgcttcctgaggctggat
IL‐4	atcatcggcattttgaacgaggtc	accttggaagccctacagacga
IL‐17	cagactacctcaaccgttccac	tccagctttccctccgcattga
Foxp3	cctggttgtgagaaggtcttcg	tgctccagagactgcaccactt
RORγt	gtggagtttgccaagcggcttt	cctgcacattctgactaggacg
T‐bet	ccacctgttgtggtccaagttc	ccacaaacatcctgtaatggcttg
β‐actin	gatggtgaaggtcggtgtga	tgaacttgccgtgggtagag

Abbreviations: INF, interferon; RORγt, retineic‐acid‐receptor‐related orphan nuclear receptor gamma t; TGF, transforming growth factor.

### 2.6 Lentiviral infection

The mouse IL‐23R lentiviral vector and corresponding control vector (pLenti‐GIII‐CMV‐GFP^−^2A‐Puro) were obtained from Applied Biological Materials Inc (Cat# 248680640395). The lentiviruses were packaged, purified, and titrated by Wuhan Atagenix Biotech Inc. Splenic CD4^+^CD25^+^ Treg‐enriched cells were sorted from normal wild‐type C57BL/6 male mice using the EasySep™ Mouse CD4^+^CD25^+^ Regulatory T Cell Isolation Kit (Stemcell, Cat# 18783) following the supplier's manual. CD4^+^CD25^+^ T cells were suspended at a density of 1 × 10^6^/ml in RPMI1640 supplemented 10% FCS, 1 µg/ml soluble anti*‐*CD28 antibody (BioLegend, Cat# 102101), 2 ng/ml mouse TGF‐β1 (BioLegend, CAT# 763102) and 10 ng/ml mouse IL‐2 (BioLegend, Cat# 575409). These cells were seeded into a 24‐well plate precoated with 5 µg/ml plate‐bound anti*‐*CD3 antibody (BioLegend, Cat# 145‐2C11). Polybrene (Sigma‐Aldrich, Cat# TR^−^1003) was added into the culture at a final concentration of 5 µg/ml. The lentiviral particles were then added to the culture at the multiplicity of infection of 10 to incubated Tregs for 18 h at 37°C. The supernatant was then replaced by fresh media containing the same stimuli and Tregs were incubated for additional 2 days. GFP^+^ cells (i.e., successfully infected Tregs) were quantified and sorted by flow cytometry for further tests. The expression of Fopx3, CD25, and IL‐23R in lentivirus‐infected Tregs was detected by flow cytometry as described previously.

### 2.7 In vitro cell culture

Splenic CD4^+^CD25^‐^ conventional T cells were sorted from wild‐type mice by FACS and labeled with 5 µM cell proliferation dye eFluor™ 670 (ThermoFisher, Cat# 65‐0840‐85) following the vendor's instructions. 1 × 10^5^ labeled conventional T cells and 1 × 10^5^ Tregs were seeded in a 96‐well culture plate precoated with the anti‐CD3 antibody, in the presence of 1 µg/ml soluble anti‐CD28 antibody and 10 ng/ml IL‐2. On day 2 after coculture, GFP^‐^ cells, that is, conventional T cells, were sorted by flow cytometry to quantify cytokine expression. On day 5 after coculture, the dilution of eFluor™ 670 was measured by flow cytometry to compare the proliferation of conventional T cells.

To determine the effect of IL‐23 on IL‐23R^−^overexpressing Tregs, 1 × 10^6^/ml lentivirus‐infected Tregs were treated with the above‐mentioned agonistic antibodies in the presence of 10 ng/ml mouse IL‐2 and 20 ng/ml IL‐23 (BioLegend, Cat# 589002) for 24 h. Cells were then restimulated with 50 ng/ml PMA and 1 μg/ml ionomycin in the presence of 2.5 mg/ml brefeldin A and 2 μM monensin for 3 h, followed by intracellular cytokine staining.

### 2.8 Adoptive transfer

OSCC‐bearing wild‐type C57BL/6J mice were anesthetized by isoflurane inhalation. 2 × 10^6^ lentivirus‐infected Tregs were suspended in 100 µl of saline and infused into each mouse via retro‐orbital injection. One week later, the recipients were euthanized and their tongues were harvested to analyze exogenous Tregs and 4‐NQO‐induced lesions.

### 2.9 Tongue tissue histology

Tongues were excised, immersed in 10% neutral buffered formalin (Sigma‐Aldrich, Cat# HT501128‐4L) for 1 day, longitudinally bisected, and embedded in paraffin. Consecutive 5‐μm sections were prepared and stained with hematoxylin (Sigma‐Aldrich, Cat# H3136) and eosin (Sigma‐Aldrich, Cat# 212954) following the standard procedures. Slides were recorded on a Leika DMi1 optical microscope and analyzed on ImageJ. The lesion incidence was evaluated based on reported criteria.^7^, ^26^ Briefly, each section was scored according to the most severe lesion present and categorized into the following grades: normal epithelium, hyperkeratosis, dysplasia, or squamous cell carcinoma (SCC). To analyze lesion burden, the perimeters of normal epithelium, hyperkeratosis, dysplasia, or SCC were measured and calculated as the percentages of the total perimeter of the tongue section.

### 2.10 Statistical analysis

Each experiment was repeated two or three times. The data were expressed as mean ± SEM. Student's *t*‐test or one‐way analysis of variance with Fisher PLSD post hoc test was applied to compare mean values among different groups. A *p* value less than .05 was significant.

## 3 RESULTS

### 3.1 A treg subpopulation expresses IL‐23R in the tongue after OSCC induction

**Figure 1:**
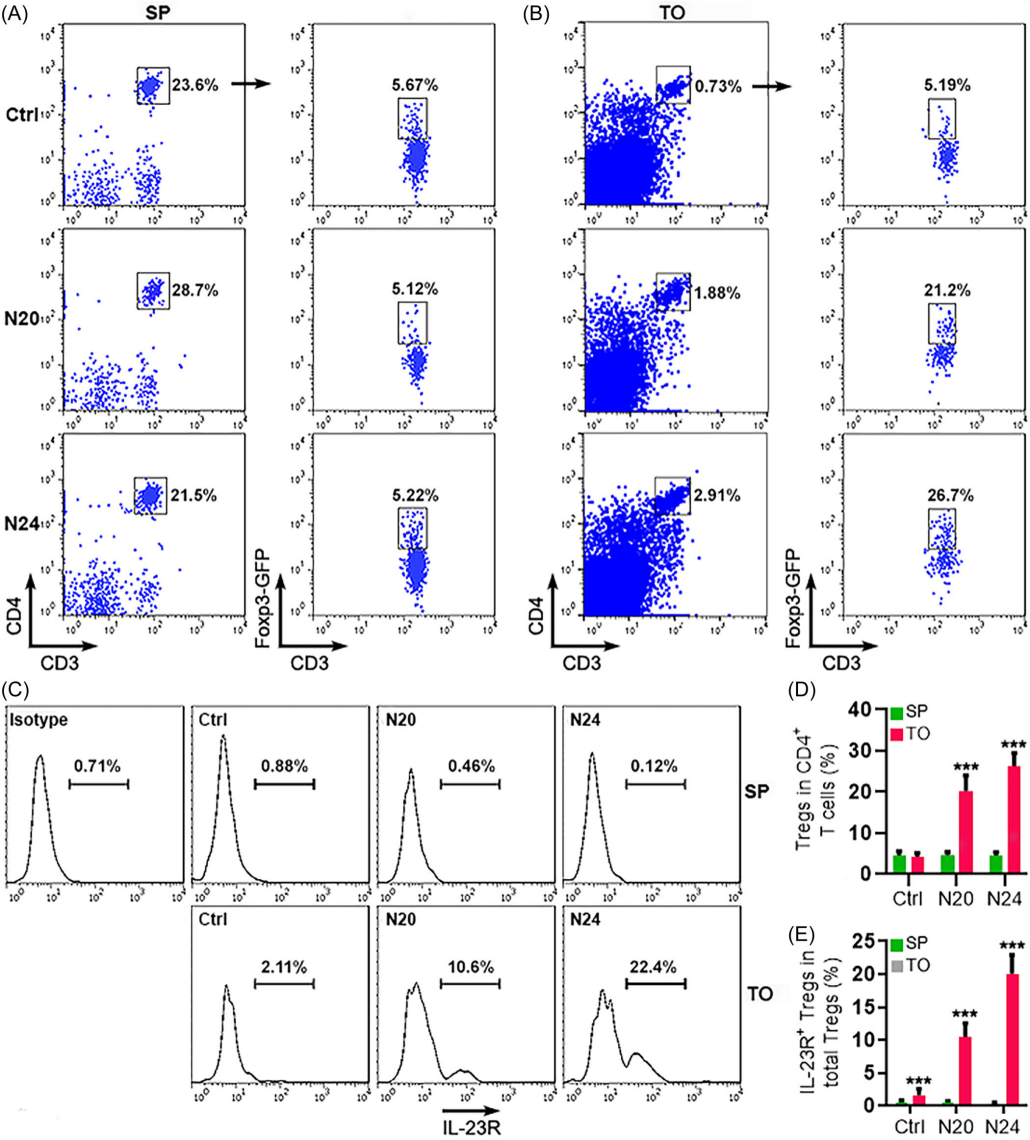
Detection of IL‐23R on Tregs. (A) Recognition of Tregs in the spleens (SP) of control mice fed with sterile water (Ctrl), mice at week 20 after 4‐NQO exposure (N20), and mice at week 24 after 4‐NQO exposure (N24). CD3^+^CD4^+^ T cells were gated in live mononuclear cells, and Foxp3‐GFP^+^ Tregs were subsequently gated in CD3^+^CD4^+^ T cells. (B) Recognition of Tregs in the tongues (TO). The gating strategy is the same as (A). (C−E) IL‐23R expression on Foxp3‐GFP^+^ Tregs. The representative histograms are displayed in (C). The statistics of the frequencies of total Tregs and IL‐23R^+^ Tregs are shown in (D, E). Isotype: isotype control. *N* = 6 mice per group. ***p < .001. Student's *t*‐test.

OSCC was induced in Foxp3‐GFP mice by drinking 4‐NQO‐containing water for 18 weeks followed by drinking sterile water for 2 (i.e., 20 weeks after initial 4‐NQO administration) or 6 weeks (i.e., 24 weeks after initial 4‐NQO administration) (Supporting Information: Figure 2). Mononuclear cells were then harvested from the spleens and tongues of these mice. The spleens were used as a control organ for the cancerous tongues. After excluding dead cells by propidium iodide staining (Supporting Information: Figure 3), CD3^+^CD4^+^ T cells were recognized in the mononuclear cells and then CD4^+^GFP^+^ Tregs were defined (Figure 1A,B). After OSCC induction, Treg frequency was remarkably increased in the tongue relative to the spleen (Figure 1D). Analysis of IL‐23R staining indicated no IL‐23R expression on splenic CD4^+^GFP^+^ Tregs (Figure 1C,E). CD4^+^GFP^+^ Tregs in the tongue, however, had a significant IL‐23R^−^ expressing subpopulation, especially after OSCC induction. IL‐23R^−^expressing Tregs accounted for around 10% and 20% of CD4^+^GFP^+^ Tregs at week 20 and week 24 after initial 4‐NQO administration, respectively (Figure 1C,E).

### 3.2 IL‐23R^+^ tregs have Th1‐like property

**Figure 2:**
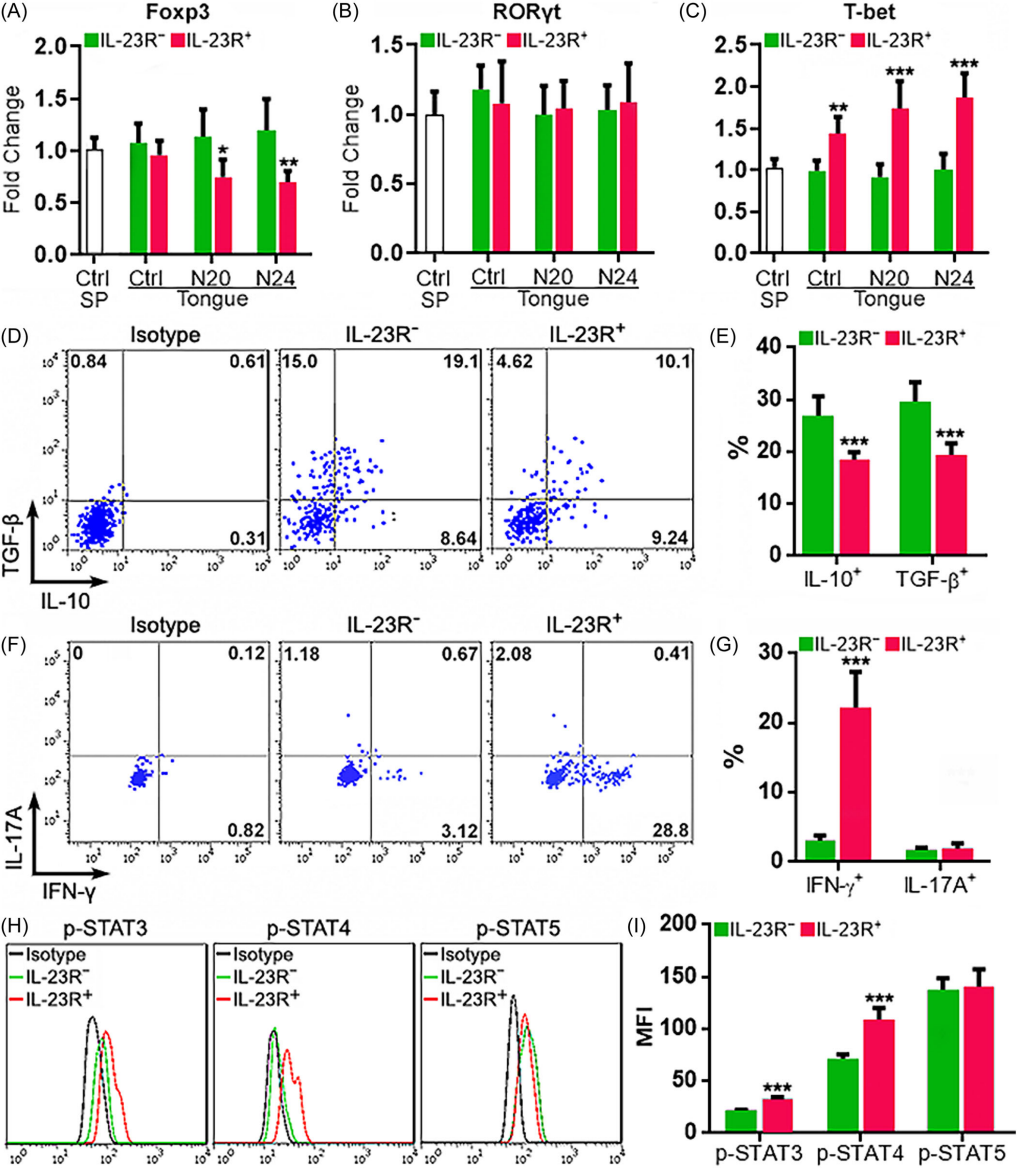
Functional properties of infiltrating IL‐23R^−^Tregs and IL‐23R^+^ Tregs. (A−C) Relative mRNA abundances of *Foxp3*, *Rorc*, and *Tbx21* in Treg subpopulations in the tongues of control mice (Ctrl), mice at Week 20 after 4‐NQO exposure (N20), and mice at Week 24 after 4‐NQO exposure (N24). The whole splenic Tregs sorted from normal mice (Ctrl SP) were used as an expression control for each master regulator. IL‐23R^−^: IL‐23R^−^Tregs. IL‐23R^+^: IL‐23R^+^ Tregs. (D) Representative dot plots showing the intracellular staining of IL‐10 and TGF‐β in Tregs at Week 24 after 4‐NQO exposure. (E) Statistics of the frequencies of Tregs expressing IL‐10 and TGF‐β. (F) Representative dot plots showing the intracellular staining of IFN‐γ and IL‐17A in Tregs at Week 24 after 4‐NQO exposure. (G) Statistics of the frequencies of Tregs expressing IFN‐γ and IL‐17A. (H) Representative histograms indicating the activating phosphorylation of STAT3, STAT4, and STAT5 in Tregs at Week 24 after 4‐NQO exposure. (I) Statistics of the mean fluorescence intensities of phosphorylated STATs in (H). *N* = 5 mice per group. *p < .05; **p < .01; ***p < .001. Student's *t*‐test. INF, interferon; STAT, signal transducer and activator of transcription; TGF, transforming growth factor.

The IL‐23R^−^ and IL‐23R^+^ Treg subpopulations were sorted from the tongues by flow cytometry for further analysis (Supporting Information: Figure 4). First, the expression of master regulators key to Treg, Th17, and Th1 differentiation was assessed by quantitative RT‐PCR. The whole splenic Tregs sorted from normal mice were used as an expression control for each master regulator. As shown in Figure 2A, IN normal control mice, splenic Tregs, tongue IL‐23R^−^ Tregs, and tongue IL‐23R^+^ Tregs expressed comparable levels of Foxp3 mRNAs. After OSCC induction, Foxp3 mRNAs were not significantly changed in tongue IL‐23R^−^ Tregs but downregulated in tongue IL‐23R^+^ Tregs at week 20 and 24. Tongue IL‐23R^−^ Tregs and IL‐23R^+^ Tregs always expressed the same basal levels of retineic‐acid‐receptor‐related orphan nuclear receptor gamma t (RORγt) mRNAs as normal splenic Tregs (Figure 2B). Interestingly, tongue IL‐23R^−^ Tregs, regardless of OSCC induction or not, always expressed the same levels of T‐bet mRNAs as normal splenic Tregs. However, tongue IL‐23R^+^ Tregs, regardless of OSCC induction or not, always expressed higher T‐bet mRNAs than tongue IL‐23R^−^ Tregs (Figure 2C). Because IL‐23R^+^ Tregs at week 24 were more abundant, they were analyzed in the following experiments. Flow cytometry analysis revealed that RORγt protein was not significantly expressed in either IL‐23R^−^ or IL‐23R^+^ Tregs. T‐bet was slightly expressed in tongue IL‐23R^−^ Tregs but remarkably upregulated in tongue IL‐23R^+^ Tregs (Supporting Information: Figure 5). Intracellular cytokine staining revealed that IL‐23R^−^ Tregs produced abundant IL‐10 and TGF‐β (Figures 2D,E) but very low IFN‐γ (Figure 2F,G). In contrast, IL‐23R^+^ Tregs produced lower IL‐10 and TGF‐β (Figure 2D) but remarkably higher IFN‐γ (Figure 2F,G). IL‐17A expression was almost negative (IL‐17A^+^ cells < 2.5%) in both IL‐23R^−^ Tregs and IL^−^23R^+^ Tregs (Figure 2F,G). Because IL‐23R signaling is transduced by STAT3 and STAT4,^22^ the activating phosphorylation of STAT3 and STAT4 in the two subpopulations was assessed. Phosphorylation of STAT3 and STAT4 was higher in IL‐23R^+^ Tregs relative to IL‐23R^−^ Tregs (Figures 2H,I). STAT5 phosphorylation, which is crucial to Treg differentiation and function, was equivalent in these subpopulations (Figures 2H,I). Additionally, the expression of IL‐4, IL‐5, and phosphorylated STAT6 (Tyr641) was negative in both IL‐23R^‐^ and IL‐23R^+^ Tregs, indicating no signs of Th2 polarization of the two subsets (Supporting Information: Figure 6).

**Figure 3:**
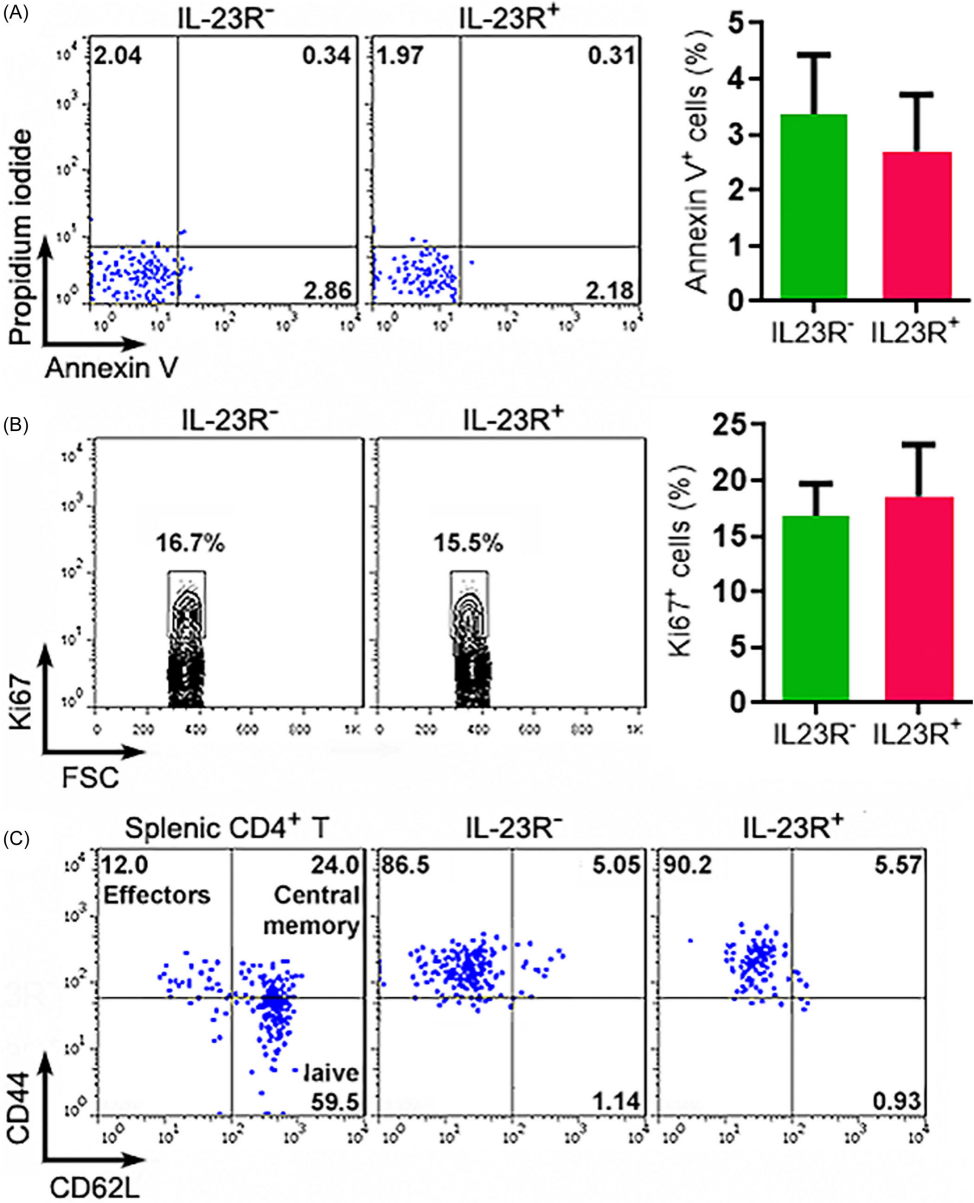
Apoptosis, proliferation, and activation status of Treg subpopulations at Week 24 after 4‐NQO exposure. (A) Treg apoptosis. Left panel: representative dot plots. Right panel: statistics of Annexin V^+^ Tregs. IL‐23R^‐^: IL‐23R^‐^ Tregs. IL‐23R^+^: IL‐23R^+^ Tregs. (B) Measurement of Treg proliferation by Ki67 staining. Left panel: representative contour plots. Right panel: statistics of Ki67^+^ Tregs. (C) Treg activation status based on the expression of CD44 and CD62L. Splenic CD4^+^ T cells were used to tell naive and effector T cells. The images represent two independent experiments. *N* = 5 mice per group. Student's *t*‐test.

The viability of the two Treg subpopulations was almost the same, as evidenced by similar proportions of apoptotic cells (Figure 3A). IL‐23R^−^ Tregs and IL‐23R^+^ Tregs had comparable amounts of Ki67^+^ cells, suggesting they proliferated at the same rate (Figure 3B). Meanwhile, the majority of either IL‐23R^−^ Tregs or IL‐23R^+^ Tregs were CD44^+^CD62L^−^, implying that they were activated effector T cells (Figure 3C).

### 3.3 IL‐23R^+^ tregs are less immunosuppressive than IL‐23R^‐^ tregs

**Figure 4:**
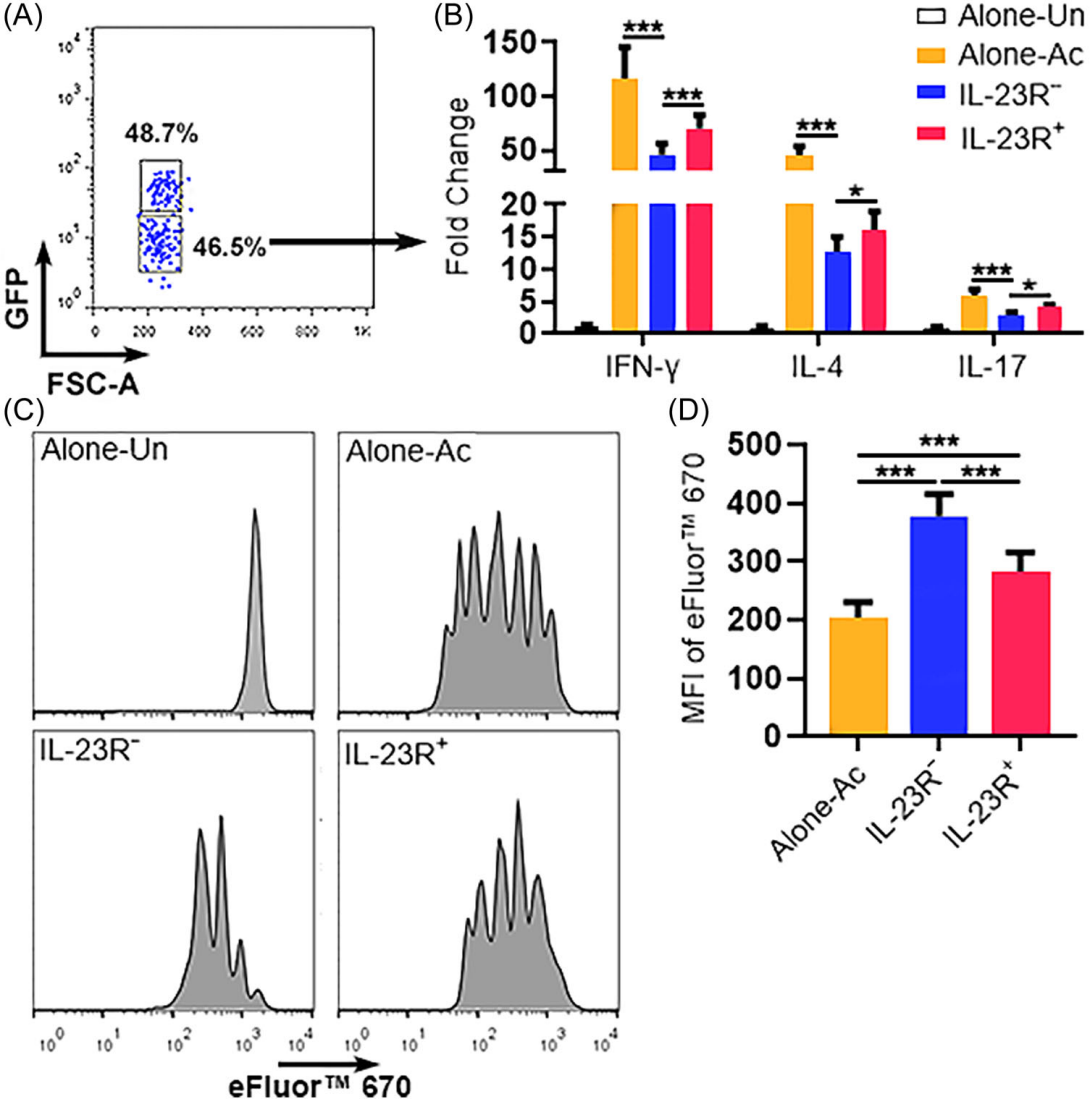
The immunosuppressive function of Treg subpopulations. (A) Sorting GFP^−^ conventional T cells after 2‐day coculture with IL‐23R^+^ Tregs. The same sorting strategy was applied after coculture with IL‐23R^−^Tregs. (B) Relative mRNA abundances of indicated cytokines in GFP^−^ conventional T cells. Alone‐Un: Unstimulated conventional T cells alone. Alone‐Ac: Activated conventional T cells alone. IL‐23R^−^: Activated conventional T cells cocultured with IL‐23R^−^ Tregs. IL‐23R^+^: Activated conventional T cells cocultured with IL‐23R^+^ Tregs. (C) Representative histograms showing eFluor™ 670 dilution in conventional T cells. (D) Statistics of the mean fluorescence intensities of eFluor™ 670 in (C). *N* = 6 mice per group.*p < .05; ***p < .001. One‐way ANOVA. ANOVA, analysis of variance; IL‐23R, interleukin‐23 receptor; mRNA, messenger RNA.

To evaluate the immunosuppressive function of Treg subpopulations, splenic CD4^+^CD25^−^ conventional T cells were enriched from normal mice, labeled with eFluor™ 670, and cocultured with IL‐23R^−^ Tregs or IL‐23R^+^ Tregs in the presence of agonistic antibodies against CD3 and CD28. On day 2 after coculture, GFP^−^ conventional T cells were sorted to quantify the mRNA levels of IFN‐γ, IL‐4, and IL‐17 (Figure 4A). As shown in Figure 4B, activated conventional T cells increased the expression of these cytokines especially IFN‐γ and IL‐4. IL‐23R^−^ Tregs remarkably inhibited the upregulation of these cytokines, whereas IL‐23R^+^ Tregs were less competent to do so. Next, the proliferation of conventional T cells was measured by eFluor™ 670 dilution on Day 5 after coculture. IL‐23R^−^ Tregs profoundly suppressed the division of conventional T cells while IL‐23R^+^ Tregs were less effective in doing so (Figure 4C,D). Therefore, IL‐23R^+^ Tregs were less immunosuppressive than IL‐23R^−^ Tregs.

### 3.4 In vitro IL‐23R overexpression affects Treg function in the presence of IL‐23

**Figure 5:**
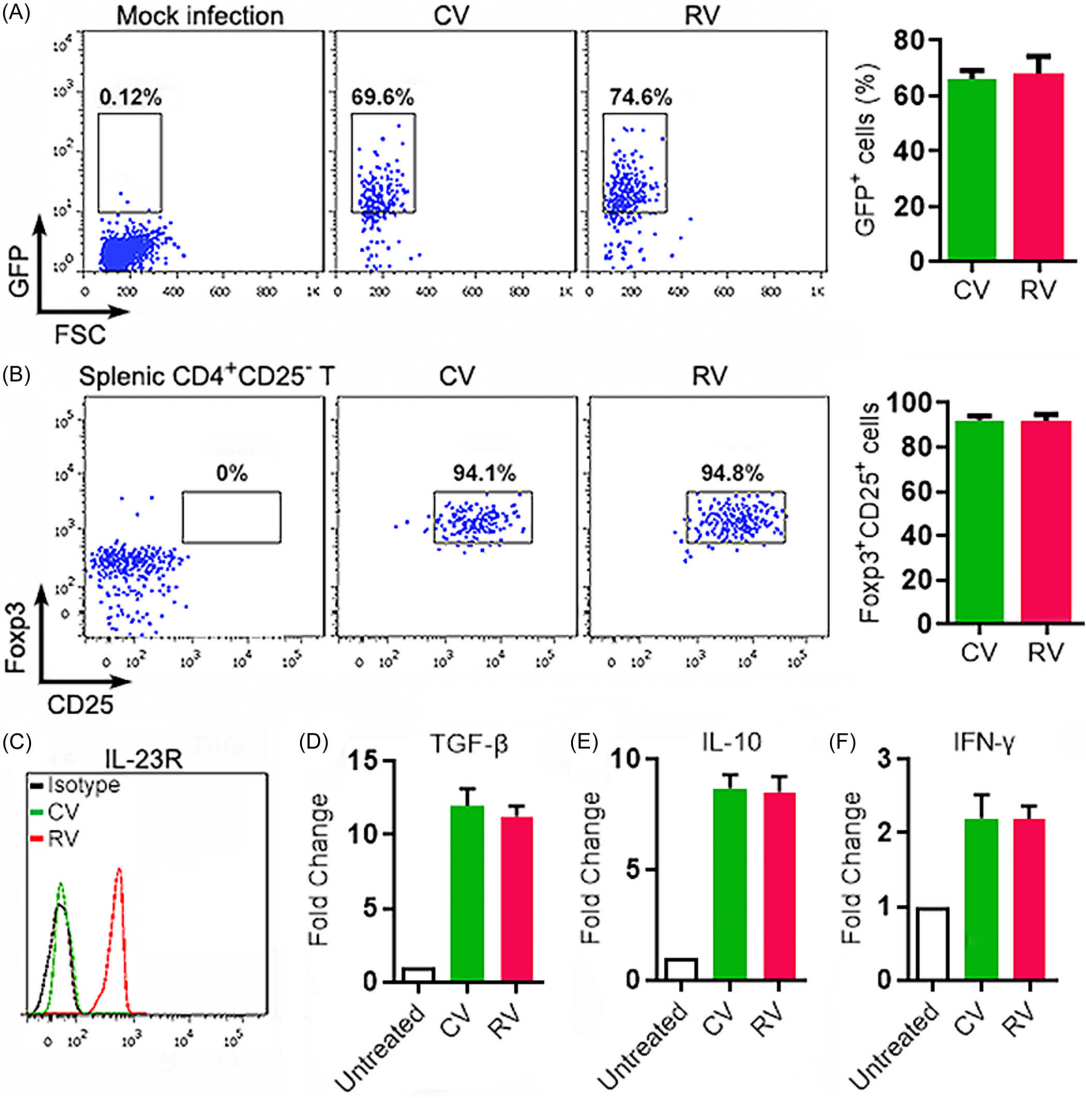
Treg phenotype and function after lentiviral infection. (A) GFP expression after lentiviral infection of wild‐type splenic CD3^+^CD4^+^CD25^+^ Treg‐enriched cells. Left panel: representative dot plots. Right panel: statistics of GFP^+^ cell frequencies. Mock infection: without lentivirus. CV: infection with control virus. RV: infection with IL‐23R‐encoding virus. (B) Expression of Foxp3 and CD25 in infected Tregs. Primary splenic CD4^+^CD25^−^ T cells were used as a negative control. Left panel: representative dot plots. Right panel: statistics of Foxp3^+^CD25^+^ cells after infection. (C) IL‐23R expression on infected Tregs. The image represents two independent experiments. (D−F) Relative mRNA abundances of indicated cytokines in lentivirus‐infected Tregs. Untreated: untreated Tregs. *N* = 3 samples per group. ***p < .001. Student's *t*‐test. IL‐23R, interleukin‐23 receptor.

To evaluate the impact of IL‐23R on Treg function, splenic CD3^+^CD4^+^CD25^+^ Treg‐enriched cells were sorted from wild‐type mice and stimulated with agonistic antibodies in the presence of TGF‐β. Stimulated Tregs were then infected with the control lentivirus (CV) or IL‐23R‐encoding lentivirus (RV) overnight. Because the lentivirus contained a GFP sequence, the infection efficiency was monitored based on the proportion of GFP^+^ Tregs on Day 2 after infection. Around 70% of Tregs became GFP^+^ after infection with either CV or RV (Figure 5A). The expression of Foxp3 and CD25 were comparable in CV‐infected and RV‐infected Tregs (Figure 5B). IL‐23R overexpression on RV‐infected Tregs was confirmed by flow cytometry (Figure 5C). CV‐infected and RV‐infected Tregs expressed comparable TGF‐β, IL‐10, and IFN‐γ (Figures 5D,F). Therefore, in vitro IL‐23R overexpression did not alter Treg immunosuppressive function in the absence of IL‐23 (IL‐23R ligand).

**Figure 6:**
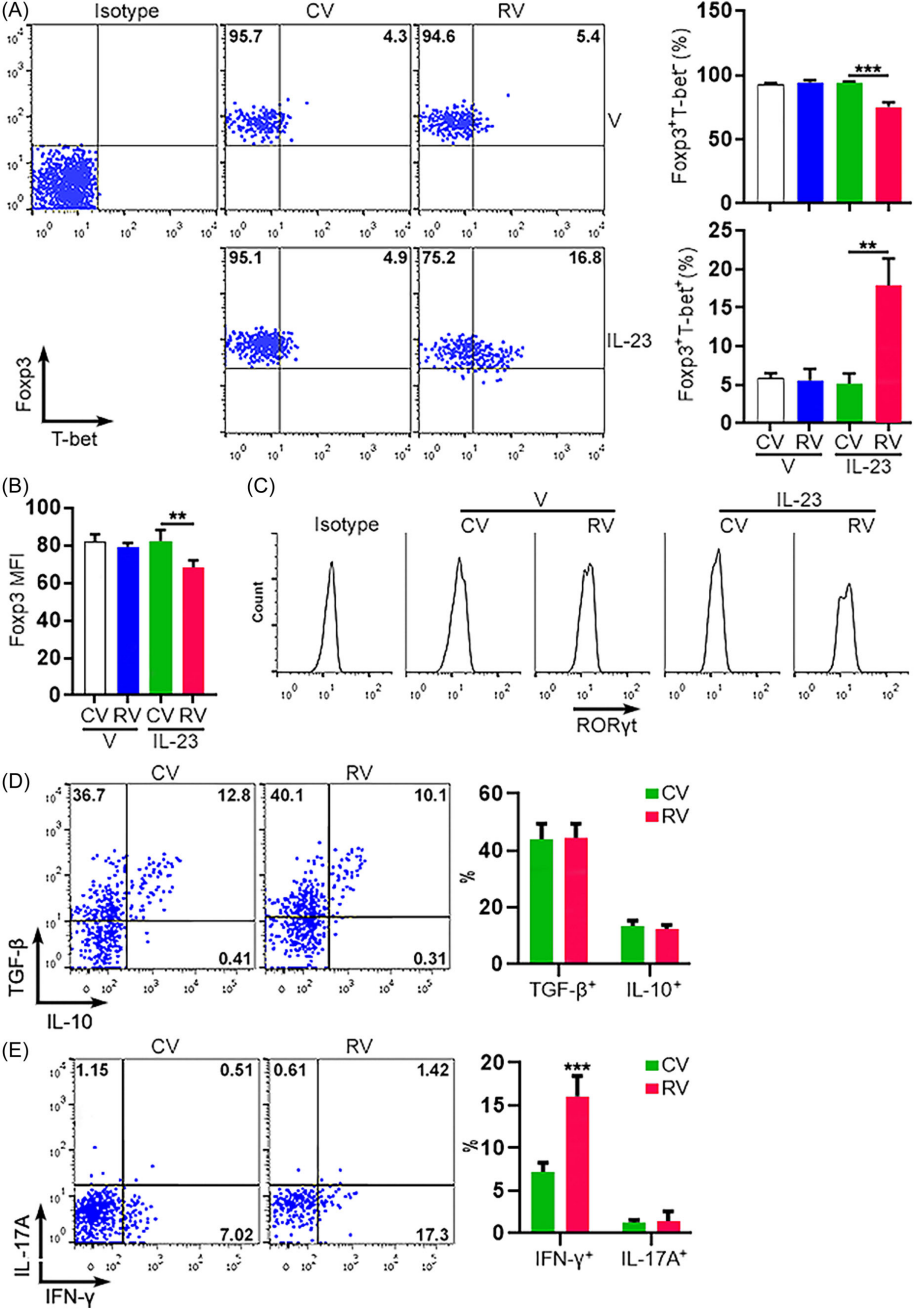
The expression of master regulators and cytokines in lentivirus‐infected Tregs after IL‐23 treatment. (A) Costaining of Foxp3 and T‐bet in Tregs after treatment with PBS (V) or IL‐23. Left panel: representative dot plots. Right panel: statistics. CV: infection with control virus. RV: infection with IL‐23R‐encoding virus. (B) The mean fluorescence intensities of Foxp3 in Tregs after treatment with PBS (V) or IL‐23. (C) Representative histograms of RORγt staining in Tregs after treatment with PBS (V) or IL‐23. The data represent two independent experiments. (D, E) Intracellular staining of TGF‐β/IL‐10 (D) and IFN‐γ/IL‐17A (E) in lentivirus‐infected Tregs after IL‐23 treatment and restimulation. Left panels: representative dot plots. Right panels: statistics of the frequencies of cytokine‐expressing Tregs. *N* = 5 or 6 samples per group. **p < .01; ***p < .001. Student's *t*‐test. IL‐23R, interleukin‐23 receptor; INF, interferon; PBS, phosphate‐buffered saline; TGF, transforming growth factor.

To determine the effect of IL‐23 on IL‐23R‐overexpressing Tregs, lentivirus‐infected Tregs were treated with agonistic antibodies in the presence of IL‐2 and IL‐23 for 24 h. After that, the expression of Foxp3, T‐bet, and RORγt was analyzed by flow cytometry. As shown in Figure 6A, without IL‐23, about 95% of either CV‐infected Tregs or RV‐infected Tregs were Foxp3^+^T‐bet^−^, and 5% were Foxp3^+^T‐bet^+^. In the presence of IL‐23, the proportions of Foxp3^+^T‐bet^−^ cells and Foxp3^+^T‐bet^+^ cells were not significantly changed in CV‐infected Tregs. However, Foxp3^+^T‐bet^−^ cells were remarkably reduced while Foxp3^+^T‐bet^+^ cells were profoundly increased in RV‐infected Tregs in the presence of IL‐23, suggesting that the IL‐23/IL‐23R axis upregulated T‐bet expression. Importantly, the mean fluorescence intensity of Foxp3 was reduced only in RV‐infected Tregs after IL‐23 treatment, suggesting that the IL‐23/IL‐23R axis suppressed Foxp3 expression (Figure 6B **)**. Interestingly, RORγt expression was not found in all groups, suggesting that the IL‐23/IL‐23R axis did not trigger RORγt expression in this experimental setting (Figure 6C). To detect cytokine expression, after IL‐23 treatment, lentivirus‐infected Tregs were re‐stimulated with PMA and ionomycin for 3 h followed by intracellular cytokine staining. As shown in Figure 6D, CV‐infected Tregs and RV‐infected Tregs expressed comparable TGF‐β and IL‐10 after IL‐23 treatment. However, RV‐infected Tregs produced more IFN‐γ than CV‐infected Tregs after IL‐23 treatment (Figure 6E). Therefore, IL‐23 could promote IFN‐γ production while not impacting TGF‐β and IL‐10 in Tregs. IL‐17A expression was nearly negative (IL‐17A^+^ cells < 2%) in both groups (Figure 6E).

### 3.5 IL‐23R overexpression impairs treg immunosuppressive function in vivo

**Figure 7:**
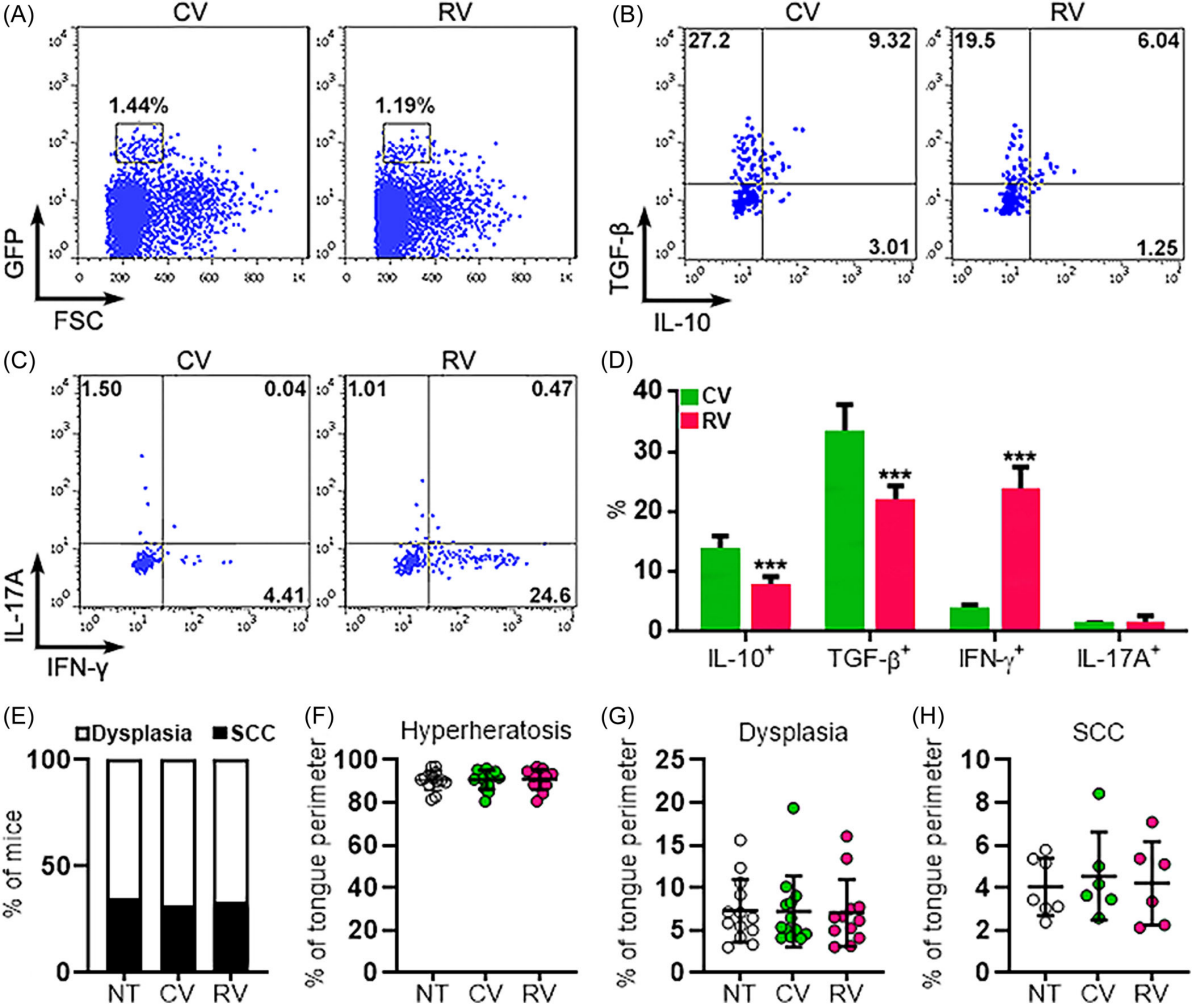
The function of lentivirus‐infected Tregs in the tongues of OSCC‐bearing mice. (A) Representative dot plots showing exogenous GFP^+^ Tregs in mononuclear cells isolated from the tongues of OSCC‐bearing recipients. CV: mice transferred with CV‐infected Tregs. RV: mice transferred with RV‐infected Tregs. (B) Representative dot plots showing intracellular staining of IL‐10 and TGF‐β in exogenous GFP^+^ Tregs. (C) Representative dot plots showing intracellular staining of IFN‐γ and IL‐17A in exogenous GFP^+^ Tregs. (D) Statistics of the frequencies of exogenous GFP^+^ Tregs expressing indicated cytokines. (E) Percentage of mice scored as indicated histology grade. (F−H) Percentage of tongue perimeter defined as hyperkeratosis (F), dysplasia (G), or SCC (H). NT: no transfer. *N* = 5 mice per group in (D). *N* = 6 or 13 mice per group in (E−H). ***p < .001. Student's *t*‐test for (D). One‐way ANOVA for (F–H). ANOVA, analysis of variance. INF, interferon; TGF, transforming growth factor.

To evaluate the effect of IL‐23R on Tregs in vivo, wild‐type C57BL/6J mice were given 4‐NQO to induce OSCC. Twenty‐four weeks after the start of OSCC induction, lentivirus‐infected Tregs were adoptively transferred into OSCC‐bearing wild‐type mice (Supporting Information: Figure 7). One week after transfer, GFP^+^ Tregs, i.e. exogenous Tregs, were found in mononuclear cells isolated from the tongues of the recipients (Figure 7A). The frequencies of CV‐infected Tregs and RV‐infected Tregs were comparable, suggesting that Treg transmigration and survival were not impacted by IL‐23R expression (Figure 7A). Consistent with the in vitro results, RV‐infected Tregs expressed significantly less IL‐10 and TGF‐β but higher IFN‐γ than CV‐infected Tregs (Figures 7B−D). IL‐17A expression was almost negative (IL‐17A^+^ cells < 2%) in both CV‐infected Tregs and RV‐infected Tregs (Figure 7C,D). However, the incidence and burden of dysplasias and OSCC were not significantly different between the mice receiving CV‐infected Tregs and the mice receiving RV‐infected Tregs (Figures 7E,H), suggesting that exogenous Tregs cells were unable to impact the development of 4‐NQO‐induced lesions. A better animal model will be required to further elucidate the role of the two Treg subpopulations in OSCC.

### 3.6 IL‐23 is produced by OSCC microenvironment cells

**Figure 8:**
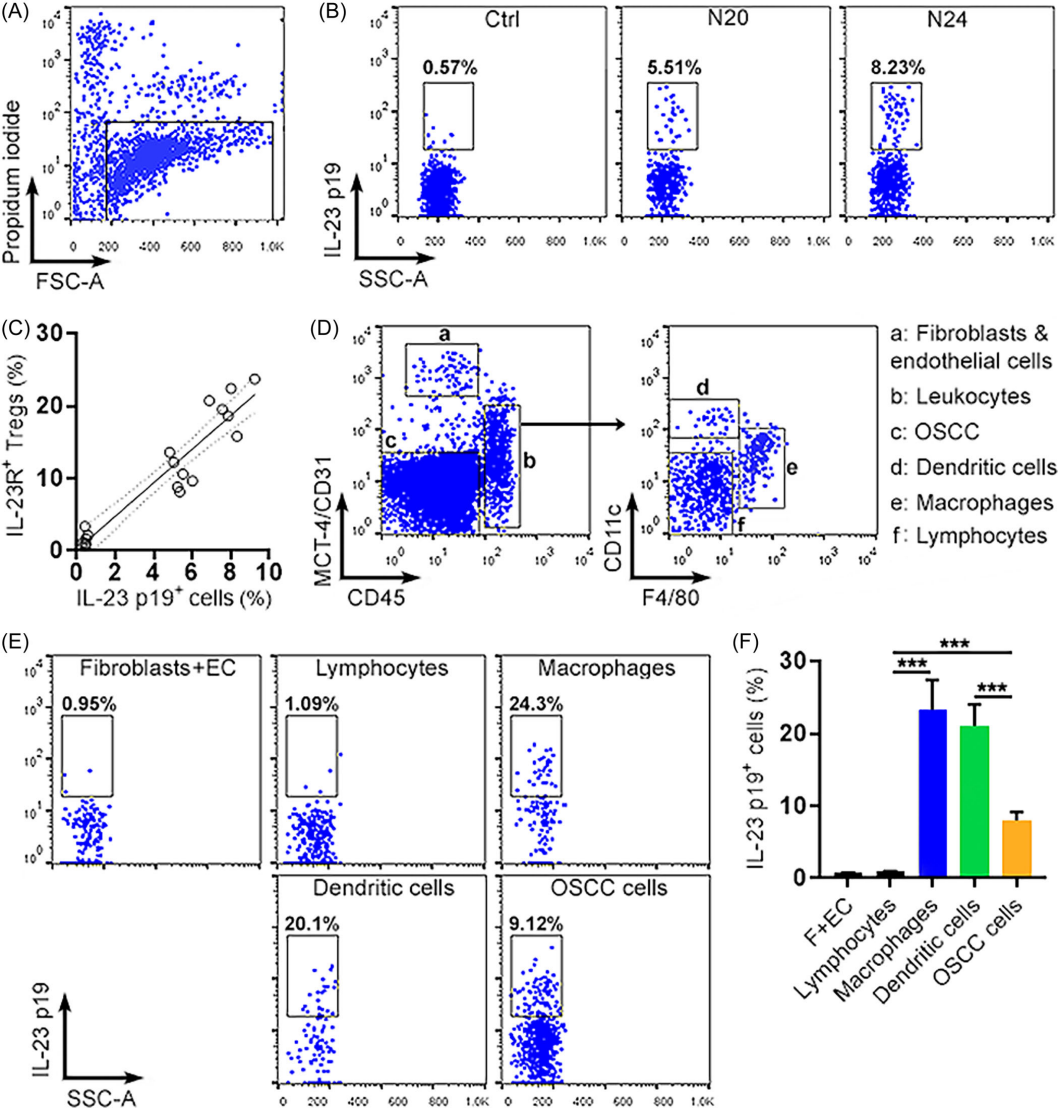
IL‐23 p19 expression in the OSCC microenvironment. (A) A representative dot plot showing live tongue cells after excluding propidium iodide‐positive dead cells. (B) Dot plots showing IL‐23 p19 expression in the whole live tongue cells in normal mice (Ctrl), mice at week 20 (N20) and week 24 (N24) after 4‐NQO exposure. (C) Correlation between the proportions of IL‐23 p19^+^ tongue cells and the proportions of IL‐23R^+^ Tregs. *N* = 6 mice per group. (D) Dot plots showing distinct cell types in isolated tongue cells at week 24. (E and F) IL‐23 p19 expression in indicated cell types. Representative dot plots are shown in (E). Statistics of the proportions of IL‐23 p19^+^ cells in relevant cell types are shown in (F). F + EC: fibroblasts plus endothelial cells. *N* = 3 data points per group. Each data point represents cells pooled from 3 mice. ***p < .001. One‐way ANOVA. ANOVA, analysis of variance.

To find the sources of IL‐23 in the OSCC environment, we sorted live cells from mouse tongues at Week 20 and 24 after 4‐NQO exposure (Figure 8A). These cells were then subjected to intracellular staining of IL‐23 p19. As shown in Figure 8B, IL‐23 p19 expression was minute in normal tongue cells but was progressively increased at Week 20 and 24 after 4‐NQO exposure. As indicated in Figure 8C, there was a significantly positive correlation between the proportions of IL‐23 p19^+^ tongue cells and the proportions of IL‐23R^+^ Tregs (*r* = 0.9410; 95% confidence interval = 0.8456 to 0.9781; *p* < .001). To further identify IL‐23‐producing cells in malignant tongues, CD45^+^ leukocytes, MCT‐4^+^ tumor‐associated fibroblasts plus CD31^+^ endothelial cells, and CD45^−^MCT‐4^‐^CD31^−^ malignant OSCC cells were sorted from the live tongue cells by flow cytometry (Figure 8D). CD45^+^ leukocytes were further divided into CD11c^+^ dendritic cells, CD11c^−/low^F4/80^+^ macrophages, as well as CD11c^‐^F4/80^−^ lymphocytes (Figure 8D). Intracellular IL‐23 p19 staining of the above cell types indicated scarce IL‐23 p19 expression in fibroblasts, endothelial cells, and lymphocytes. More than 20% of macrophages and dendritic cells expressed IL‐23 p19, while 9% of malignant OSCC cells expressed IL‐23 p19 (Figures 8E,F). These cells might modulate the function of IL‐23R^+^ Tregs through secreting IL‐23.

## 4 DISCUSSION

In this research, we identified two Treg subpopulations in the tongues of OSCC‐bearing mice. To our knowledge, this is the first report revealing Treg heterogeneity in terms of IL‐23R expression in OSCC. An early study also described heterogeneous IL‐23R expression on tumor‐associated Tregs in a tumor implantation model.^27^ However, this study stated that IL‐23R expression leads to upregulation of Foxp3 and IL‐10.^27^ The inconsistent results of the two studies perhaps reflect sophisticated modulatory effects of distinct tumor microenvironments on Treg function. Further studies on IL‐23R^+^ Tregs in tumors especially primary tumors are therefore needed to elucidate and clarify the role of IL‐23R^+^ Tregs in tumorigenesis.

Our data show that the proportion of IL‐23R^+^ Tregs was increased from 2% to 20% after OSCC induction, suggesting that the OSCC microenvironment triggered the upregulation of IL‐23R. Although we did not reveal the factor(s) that boosted IL‐23R expression in infiltrating Tregs, it is plausible to deduce that some cytokines present in the malignant microenvironment induce IL‐23R expression. Previous and recent research indicate that IL‐6 and IL‐12 triggered IL‐23R expression on T cells.^22^ Interestingly, chronic inflammation is a common feature of OSCC and it is involved in tumor progression, invasion, and metastasis.^28^ OSCC‐associated inflammation is characterized by increased proinflammatory cytokines including IL‐6, IL‐8, TNF‐α, and other inflammatory mediators.^29^ Therefore, these cytokines likely enhance IL‐23R expression on infiltrating Tregs. Our ongoing study is investigating this possibility both in vitro and in vivo.

The role of Tregs in OSCC is controversial. Tregs lead to a poorer prognosis ^30^ or might fight OSCC development.^31^, ^32^ Such inconsistency about the role of Tregs probably arises from the heterogeneity or plasticity of Tregs. Our findings suggest that heterogeneous Tregs are present in OSCC tissues. However, the true identities and potential plasticity of these Treg subsets demand further revelation. The high expression of T‐bet and IFN‐γ suggests that the IL‐23R^+^ Tregs are mainly Th1‐like Tregs. Th1‐like Tregs have been found in hepatocellular carcinoma, colorectal cancer, and lung cancer to regulate antitumor immunity.^12^, ^13^, ^33^ In contrast, the immunosuppressive IL‐23R^−^Tregs, which produce abundant IL‐10 and TGF‐β but low IFN‐γ, likely represent classical Tregs. Collectively, the present study thus confirms and extends the heterogeneity of Tregs. In future investigations, it will be necessary to determine the fate of these Treg subsets to see whether they lose Treg identity and turn into other T helper cells.

IL‐23R pairs with the receptor molecule IL12Rβ1 to transduce IL‐23 signaling through constitutive association with transcription activator STAT3, STAT4, and JAK2 in a ligand‐dependent manner.^18^ IL‐23R^−^ related disorders include inflammatory bowel disease and psoriasis.^34^, ^35^ The IL‐23/IL‐23R axis is involved in the maintenance of Th17 cells. Interestingly, recent research demonstrated the significance of IL‐23R in the differentiation and function of Th1 and Th2 cells, suggesting the essential role of the IL‐23/IL‐23R axis in T cell‐mediated reactions.^22^, ^23^ Our data suggest that IL‐23R^+^ Tregs are likely Th1‐like cells, as evidenced by their high IFN‐γ expression and increased T‐bet expression. Other research implies that IFN‐γ and IL‐12 signaling produce Th1‐like Tregs, whereas IL‐6, IL‐21, and IL‐23 promote the formation of Th17‐like Tregs.^36^ In the future, it is necessary to use selective and combinatory cytokine knockout mice to clarify the roles of various cytokines in generating the Th1‐like function of IL‐23R^+^ Tregs.

The major limitation of this study is that the adoptive transfer of IL‐23R^+^ Tregs failed to change the development of OSCC. Perhaps the quantity of transferred Tregs was insufficient to induce a significant change in anti‐OSCC immunity, or 4‐NQO‐induced lesions were too aggressive to be inhibited by exogenous Tregs. Therefore, a better animal model will be required to elucidate the role of the two Treg subpopulations in OSCC. Treg‐specific IL‐23R knockout or transgenic mice, if available, would be applied in future investigations. Another limitation is the lack of comprehensive revelation of the phenotypic and functional differences between the two Treg subpopulations. In the future, bulk and single‐cell transcriptomics should be performed to discover the differential configurations of surface markers, signaling molecules, transcription factors, and function‐related mediators in these Tregs.

In conclusion, our data unveil distinct phenotypes and functions of heterogenous Treg subpopulations in OSCC, thus deepening the understanding of Treg response and immune tolerance in OSCC.

## AUTHOR CONTRIBUTIONS

*Conceptualization, writing – original draft preparation, writing – review and editing, funding acquisition*: Zhidan Mei. *Methodology and investigation*: Wei Li, Ning An, Mingwei Wang, and Xiguo Liu. All authors have read and agreed to the published version of the manuscript.

## CONFLICT OF INTEREST

The authors declare no conflict of interest.

## Supporting information

Supporting information.

## Data Availability

Data included in this research are available from the authors upon reasonable request and with permission from the hospital and university.
