# Supplementary material for: Interleukin‐23 receptor defines T helper 1‐like regulatory T cells in oral squamous cell carcinoma
Source: Immun Inflamm Dis. 2022 Nov 23;10(12):e746. doi: 10.1002/iid3.746 (PMC9682469; doi:10.1002/iid3.746)
Supplement: Supplementary file 1 — Supporting information. [file IID3-10-e746-s001.docx]

**Supplementary information**


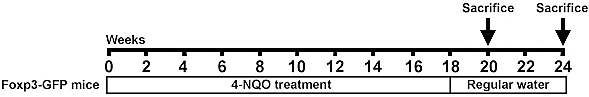


**Supplementary Figure 1.** **Schematic representation of 4-NQO treatment.**


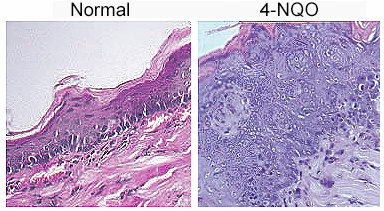


**Supplementary Figure 2.** **H&E staining of mouse tongues.** Normal: a normal mouse fed with regular water. 4-NQO: a mouse fed with 4-NQO for 18 weeks followed by water for 2 weeks. Significant OSCC lesions in the tongue were observed after the 4-NQO treatment. (Original magnification: ×100).


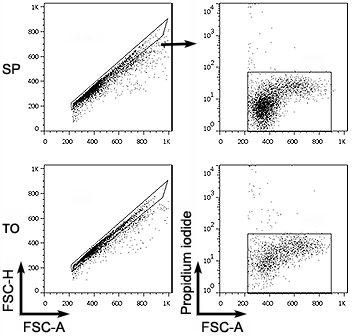


**Supplementary Figure 3.** **Single cells were gated within isolated mononuclear cells based on FSC-A and FSC-H. After that, dead cells were excluded by propidium iodide staining.** SP: spleen. TO: tongue.


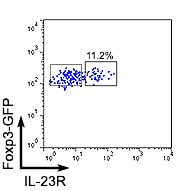


**Supplementary Figure 4.** **Gating IL-23R^+^ Tregs and IL-23R^-^ Tregs before sorting.**


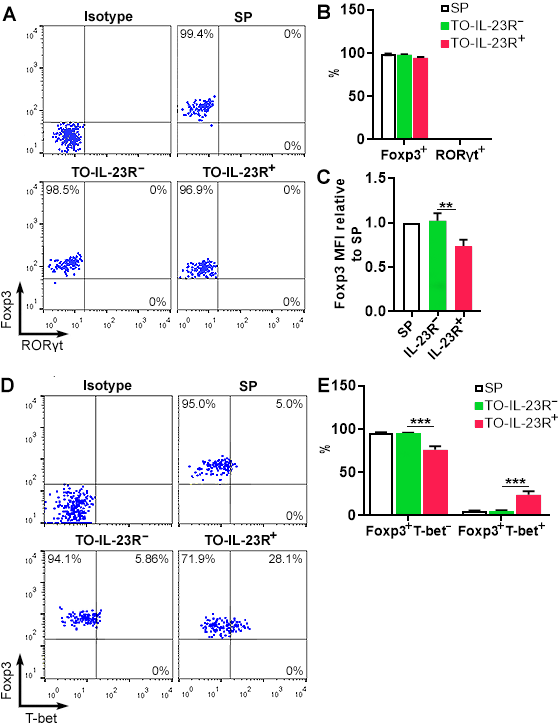


**Supplementary Figure 5.** **Protein levels of Foxp3,** **RORγt, and T-bet in Treg subsets at week 24 after 4-NQO exposure.** CD3^+^CD4^+^Foxp3-GFP^+^IL-23R^-^ and CD3^+^CD4^+^Foxp3-GFP^+^IL-23R^+^ Tregs cells were sorted from mouse tongues and subjected to intracellular staining of Foxp3, RORγt, and T-bet. **(A)** Representative dot plots showing the expression of Foxp3 and RORγt. Isotype: isotype control. SP: total splenic Tregs. TO-IL-23R^-^: tongue IL-23R^-^ Tregs. TO-IL-23R^+^: tongue IL-23R^+^ Tregs. **(B)** Statistics of the percentages of Foxp3^+^ cells and RORγt^+^ cells in each subset. Note that total splenic Tregs were tested due to the rarity of splenic IL-23R^+^ Tregs. **(C)** Foxp3 mean fluorescence intensity relative to the “SP” group. **(D)** Representative dot plots showing the expression of Foxp3 and T-bet. **(E)** Statistics of the percentages of Foxp3^+^T-bet^-^ cells and Foxp3^+^T-bet^+^ cells in each subset. N=4 mice per group in independent experiments. ***: *P*<0.001.


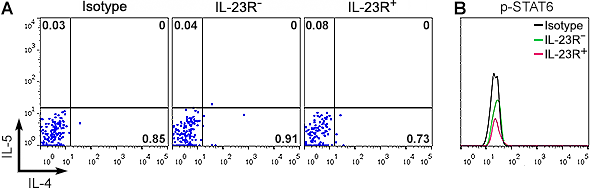


**Supplementary Figure 6. The Th2 property in infiltrating IL-23R^-^ Tregs and IL-23R^+^ Tregs. (A)** Representative dot plots showing the intracellular staining of IL-4 and IL-5 in Tregs at week 24 after 4-NQO exposure. **(B)** Representative histograms indicating the activating phosphorylation of STAT6 in Tregs at week 24 after 4-NQO exposure. The images represent three independent experiments.


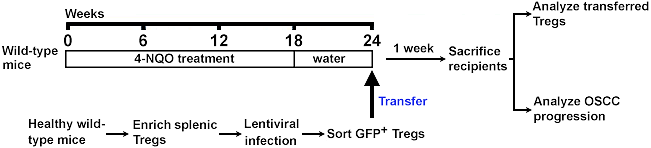


**Supplementary Figure 7. Schematic representation of the adoptive transfer assay.**
